# How to Avoid an Upside-Down Orientation of the Graft during Descemet Membrane Endothelial Keratoplasty?

**DOI:** 10.1155/2019/7813482

**Published:** 2019-08-04

**Authors:** Joanna Wasielica-Poslednik, Alexander K. Schuster, Lilian Rauch, Jessica Glaner, Aytan Musayeva, Jana C. Riedl, Norbert Pfeiffer, Adrian Gericke

**Affiliations:** Department of Ophthalmology, University Medical Center of the Johannes Gutenberg-University Mainz, Mainz, Germany

## Abstract

**Purpose:**

Incorrect anterior-posterior orientation of the Descemet endothelial complex (DEC) is one of the causes of failure of Descemet membrane endothelial keratoplasty (DMEK). We evaluated a new marking technique to avoid such a misorientation.

**Method:**

A new marking technique of the DEC was evaluated in patients requiring primary DMEK. A Braille-“R”-letter was applied dot by dot onto the stromal surface of the DEC after lifting it by injecting an air-bubble into the interface between the endothelial surface of the partially stripped graft. The positioning of the graft was intraoperatively controlled by an orientation of the Braille-“R”-letter. Laboratory tests were conducted to test the impact of the marking technique on endothelial cell count.

**Results:**

We included prospectively 37 eyes of 30 patients. Four eyes were phakic and 33 pseudophakic. Five grafts (14%) presented an undifferentiated rolling tendency in the anterior chamber, and evaluation of their positioning was possible due to orientation of the mark alone. In case of an upside-down orientation, grafts were flipped immediately. A correct orientation of the graft was achieved in all cases at the end of the surgery. The endothelial cell loss due to the mark was estimated to be less than 0.3%. At 3- and 6-month follow-ups, the mean best-corrected visual acuity was 0.21 ± 0.15 and 0.15 ± 0.11 logMAR, respectively, and endothelial cell density was 1661 ± 349 and 1618 ± 396 cells/mm^2^, respectively. Only one patient (3%) needed re-bubbling.

**Conclusions:**

To rely on the natural rolling tendency of the graft alone does not assure its correct positioning in all cases. Creation of the mark with 4 dots punctuated on the air-lifted stromal side of the DEC is a simple and endothelial cell saving marking method to ensure correct orientation of the graft during DMEK.

## 1. Introduction

In many countries, Descemet membrane endothelial keratoplasty (DMEK) became the standard treatment of hereditary or acquired corneal endothelial pathologies in recent years [1]. Quick visual rehabilitation and a reduced immunological rejection rate are the most appreciable advantages of this method. One of the preconditions for the successful DMEK procedure is a complete and gentle graft preparation. Stripping and bubble-assisted preparation techniques are the most common methods so far [2, 3].

Incorrect anterior-posterior orientation of the Descemet endothelial complex (DEC) is one of the reasons for graft detachment and failure in DMEK [4]. Many surgeons rely on the natural rolling tendency of the graft in the anterior chamber, which means that the endothelial layer faces outward from the roll. However, based on our experience, relying on the natural rolling tendency alone does not necessarily end in correct graft orientation. Different marking techniques were evaluated in order to identify the endothelial surface of the graft during DMEK. The Moutsouris sign may be helpful for identifying the orientation of the DEC in case of the endothelium-out rolling grafts only [5]. Some surgeons trephine multiple asymmetrically positioned marks on the edge of the donor graft [6–8]. However, marking the edge of the DEC with a punch results in a local loss of endothelial cells, and the lamella is more prone to get caught on intraocular structures at the punched edge or the marks are not visible when the corneal periphery is opacified. Further marking techniques imply S or F letters stamped through a stromal window created in the donor tissue [6, 9]. Moreover, recently, the use of intraoperative ocular coherence tomography (OCT) has been reported to determine the donor graft orientation [10–12]. However, such devices are not available in all operating theatres.

We developed an alternative cost and endothelial cell saving marking technique in order to avoid the upside-down positioning of the lamella and to preserve the maximum of endothelial cells and investigated the results of this technique in a prospective study.

## 2. Methods

This observational clinical study was carried out in accordance with the Declaration of Helsinki. Ethics approval was obtained from the Ethics Committee of Rhineland-Palatinate, Germany. All patients were evaluated at the Department of Ophthalmology University Medical Center of the Johannes Gutenberg University Mainz. Written informed consent was obtained from all participants. Donor tissue has been obtained from the Eye Bank of Rhineland-Palatinate, Mainz, Germany, and its use has been consented for transplantation and research. At the eye bank, the tissue has been stored at 34°C in medium 1 (Cat-No. F-9016; Biochrom, Berlin, Germany) and at least 24 hours before use in medium 2 (Cat-No. F-9017; Biochrom). Medium 2 is supplemented with 60 g Dextran 500 per 1000 ml as opposed to medium 1. Both media 1 and 2 were supplemented with gamma-irradiated fetal calf serum 10% (No. S0415, Biochrom). The inclusion criteria were male or female; >18 years of age; phakic or pseudophakic; and diagnosis of Fuchs endothelial dystrophy or corneal endothelial failure. The exclusion criteria were previous intraocular surgery except for uncomplicated cataract surgery.

### 2.1. Technique

**Figure 1:**
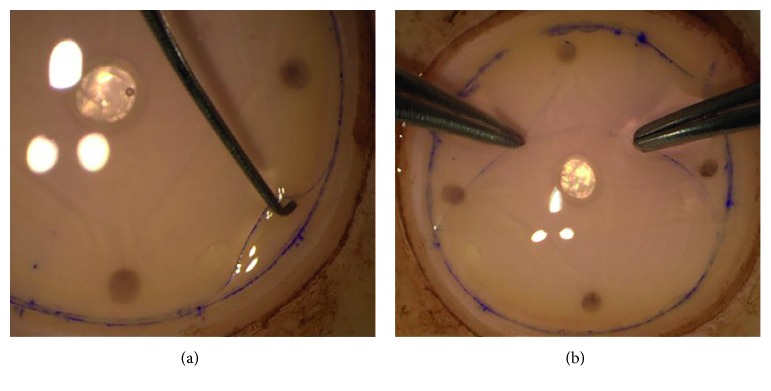
The Descemet endothelial complex of 9.5 mm in diameter is detached circularly from the peripheral corneal tissue (a) and peeled with two tweezers using different peeling sites towards the center of the cornea (b).

All graft preparations and DMEK surgeries were performed by the same experienced surgeon (AG). The graft preparation was performed immediately before the surgery using the stripping technique. A cornea was centered on the punch base using suction (Barron Vacuum Donor Cornea Punch, Katena Products, Inc., Denville, NJ, USA). A superficial cut of the DEC was performed using a 9.5 mm donor trephine. The DEC was detached circularly from the peripheral corneal tissue using a Sinskey hook (Geuder AG, Heidelberg, Germany) and peeled longitudinally in order to avoid stress lines towards the center of the cornea using straight pointed tweezers (Geuder) (Figures 1(a) and 1(b). The DEC was not stripped completely but left attached at the center. The tissue was kept moist with a few drops of the storage medium. Staining with RS-Blue (AL.CHI.MI.A. Srl, based in Ponte San Nicolò, Padova, Italy) was used in challenging cases only.

**Figure 2:**
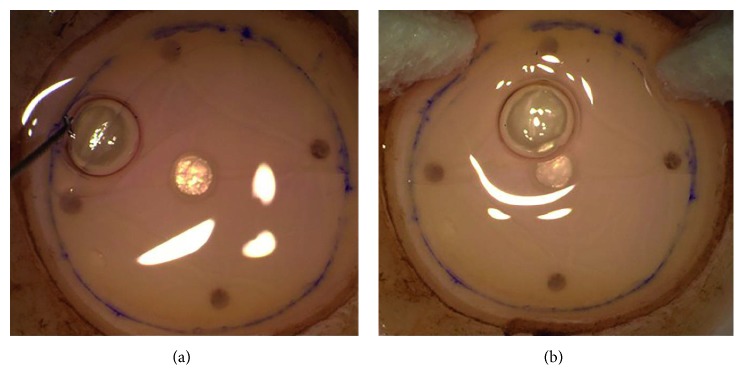
A peeled half of the lamella is folded down and a small air bubble is injected with a 30 G needle between the endothelial surfaces of the graft (a); the stromal surface of the lamella covering the air-bubble is dried with triangle swabs (b).

As soon as half of the lamella was peeled off and folded down, a small air bubble was injected with a 30 G needle connected to a 1 ml syringe into the interface between the endothelial surfaces of the graft. The stromal surface of the lamella covering the air bubble was dried with triangle swabs (Merocel®, Beaver-Visitec International, Inc., Waltham, MA, USA) (Figures 2(a) and 2(b).

**Figure 3:**
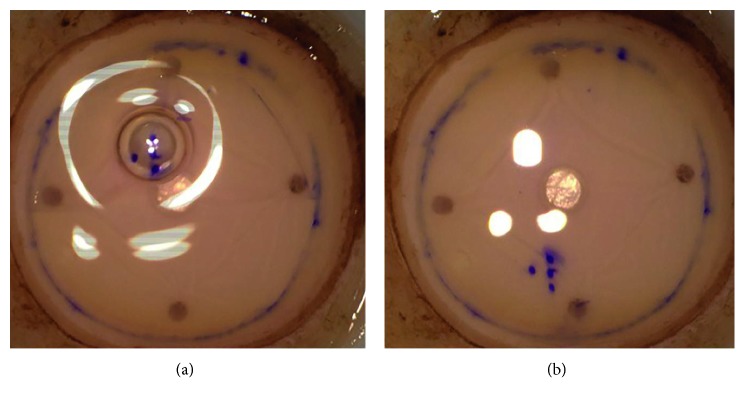
Braille-“R”-letter is punctuated with a stained Sinskey hook on the dried area (a); afterwards, the lamella is unfolded (b).

**Figure 4:**
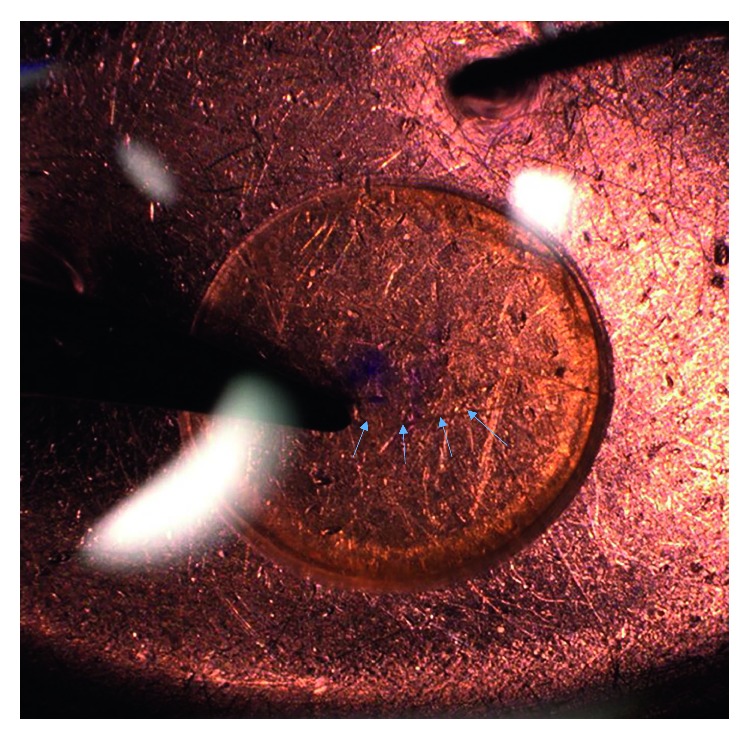
Descemet endothelial complex is peeled completely using “one touch” in culture medium bath.

The Braille-“R”-letter was applied dot by dot with a stained Sinskey hook (Secureline® skin marker) on the dried area (Figure 3(a)). Afterwards, the lamella was laid back onto the stroma (Figure 3(b)), trephined (Barron Cornea Donor Button Punch, Katena Products, Inc., Denville, NJ, USA) to the needed graft size (8.0–8.25 mm), and peeled completely using “one touch” in culture medium bath (Figure 4).

### 2.2. Laboratory Tests

Laboratory tests were conducted to test the impact of the marking technique on endothelial cell count.

Graft preparation including the marking technique was conducted on sclerocorneal buttons of nonoptical quality obtained from the Eye Bank of Rhineland-Palatinate. After preparation, the graft was placed back into medium 2 and stored at 34°C for 24 hours.

On the following day, the graft was transferred into a Petri dish and stained as described previously [13]. After a wash in phosphate-buffered saline, the graft was incubated in trypan blue dye 0.4% (GibcoTM Thermo Fisher Scientific Inc., Waltham, MA, USA) for 1 minute. The graft was then washed again in PBS before 0.5% of alizarin red dye (Santa Cruz Biotechnology, Dallas, Tx, USA) diluted in PBS at a pH of 5.0 was applied onto the graft for 4 minutes. Next, the graft was again washed in PBS, placed on a glass slide, and cover-slipped. Each graft was examined and photographed immediately after staining under a microscope (Olympus Vanox-T AH-2, Olympus Deutschland GmbH, Hamburg, Germany). Photographs of 650 × 980 *μ*m (0.637 mm^2^) were made in the area where the mark was set and in an area of corresponding localization of the opposite side of the same graft. Next, the endothelial cell number was counted and the number of endothelial cells per mm^2^ calculated.

### 2.3. Surgery

**Figure 5:**
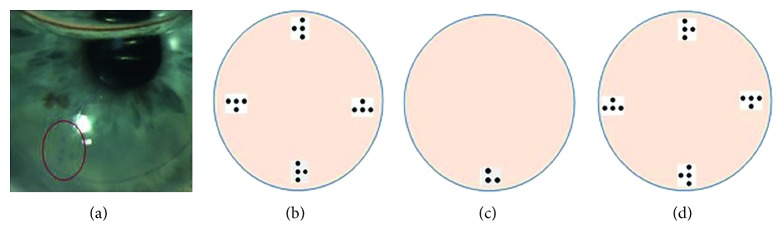
(a, b) Correct position of the Descemet endothelial complex = side point on the right (looking from the limbus); (c) correct position in case of an asymmetrical trephination; (d) upside-down position = side point on the left (looking from the limbus).

The DMEK procedure was performed under topical anesthesia (Tetracain 1% eye drops) in 35 cases and under general anesthesia in 2 cases. The descemetorhexis was performed within an area of 9.0–10.0 mm using an irrigation Descemet hook (G-38601, Geuder AG, Heidelberg, Germany) and an irrigation Descemet scraper (G-38602, Geuder AG, Heidelberg, Germany). The single-use DMEK-cartridge (G-38635, Geuder AG, Heidelberg, Germany) was used to insert the DEC into the anterior chamber. The graft was unfolded by gentle tapping of the cornea. The positioning of the graft was checked by the orientation of the Braille-“R”-letter, which was visible in all cases. An air/10% sulfur hexafluoride (SF6) bubble was injected underneath the graft as soon as the proper orientation of the Braille-“R”-letter (to the right looking from the limbus towards the center of the cornea) was achieved. In case of an upside-down positioning (orientation of the Braille-“R”-letter to the left looking from the limbus towards the center of the cornea), the graft was flipped over with a flush of balanced salt solution and the position was checked again (Figures 5(a), 5(b), and 5(d)).

The results of the marking technique were evaluated in a prospective clinical trial. The follow-up examinations were performed three months and six months postoperatively. The outcome measures were best-corrected visual acuity (BCVA) in logMAR, endothelial cell density (ECD), and re-bubbling rate. Furthermore, rolling tendencies of the graft in the anterior chamber and preparation- and surgery-time durations were documented.

## 3. Results

### 3.1. Laboratory Results

**Figure 6:**
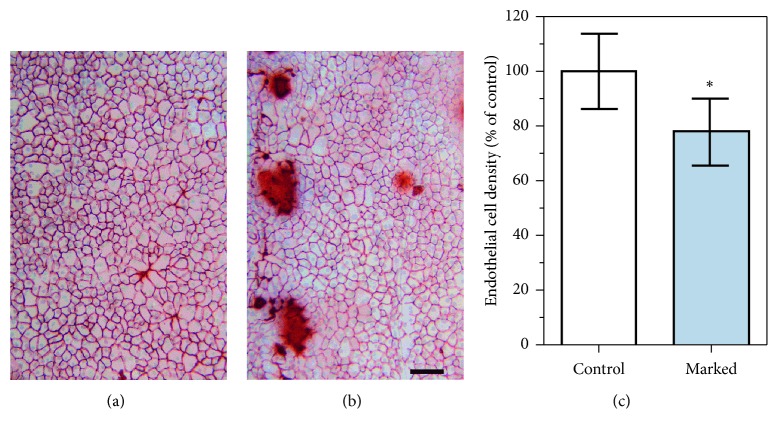
Endothelial cells were counted in areas opposite of the mark (control) (a) and in the area where the mark was set (marked) (b). Endothelial cell loss was seen only at the contact zones between the Sinskey hook and the Descemet membrane in the near vicinity (brown areas). Mean endothelial cell density was reduced in the areas where endothelial cells were counted (c). Values are presented as mean ± SD relative to control, which was set 100% (^*∗*^*P* < 0.05, *n* = 6 per group). Scale bar = 100 *μ*m.

In the areas where the marks were applied, the contact zones between the Sinskey hook and the DEC complex intensely stained with alizarin red, which can be interpreted as bare areas of the Descemet membrane (Figure 6(a) and 6(b)). However, these areas were confined to the dots only. The mean endothelial cell density in the counted areas of 0.637 mm^2^ was reduced by 22% compared to the corresponding areas of the opposite graft side (Figure 6(c)). With regard to the area of the 8.25 mm graft, which is around 53.4 mm^2^, the endothelial cell loss is estimated to be less than 0.3% and therefore negligible [14].

### 3.2. Outcome after Surgery

We included 37 eyes of 30 consecutive patients (23 females and 14 males aged 70.11 ± 11.56, range 40–85 years) requiring primary DMEK due to Fuchs endothelial dystrophy (*n* = 36) and posterior polymorphous endothelial dystrophy (*n* = 1). Four eyes were phakic and 33 pseudophakic.

The mean duration of the graft preparation was 20.9 ± 8.8 minutes (range 8–53 minutes). The marking procedure took about 2-3 minutes. The mean duration of the DMEK procedure was 24.0 ± 8.7 minutes (range 12–45 minutes). The DMEK procedure was uneventful in all cases. Intraoperatively, five grafts (14%) presented an undifferentiated rolling tendency in the anterior chamber meaning the grafts rolled with the direction of the fluid flush, and evaluation of their positioning was possible due to orientation of the mark alone. The Braille-“R”-letter mark was visible in all cases at the end of surgery. The correct orientation of the graft was achieved in all cases at the end of surgery. In the following 6 months, only one patient (3%) required re-bubbling.

The baseline BCVA was 0.44 ± 0.23 logMAR. At the 3- and 6-month follow-up visits, the mean BCVA was 0.21 ± 0.15 and 0.15 ± 0.11 logMAR, respectively.

The mean ECD of the donor corneas was 2500 ± 179 cells/mm^2^. At the 3- and 6-month follow-up, ECD was 1661 ± 349 cells/mm^2^ and 1618 ± 396 cells/mm^2^, respectively. The endothelial cell loss was 33.6% at the 3-month follow-up and 35.3% at the 6-month follow-up. Moreover, no graft decompensation and no immunological rejection were observed during follow-up.

## 4. Discussion

The new air-bubble-Braille-“R”-letter marking technique is an effective supplementation of the stripping technique used for the DEC preparation. It is a simple and cost-saving procedure. In contrast to other methods determining the donor graft orientation, it does not require any specific instruments like dermatological biopsy punch or intraoperative OCT [7–12].

Many surgeons rely on the natural endothelium-out orientation of the graft roll in the anterior chamber alone. However, the orientation of the DEC may vary depending on the donor's age, endothelial cell density, or its elastic properties [15]. In the present study, we found that none of the donor grafts spontaneously rolled with the endothelial side inwards but 14% of the donor grafts presented an undifferentiated rolling tendency, which means that the grafts rolled with the direction of the fluid flush and orientation could only be achieved by observation of the mark. The correct orientation of the DECs marked with the air-bubble-Braille-“R”-letter technique was achieved in all patients at the end of the primary DMEK procedure. At the 6-month follow-up, only one eye required re-bubbling and none of the patients required repeated DMEK. Of note, our re-bubbling rate was very low compared to rates between 5.4% and 23% reported in other studies [16, 17].

The need for establishing marking techniques enabling reliable recognition of the endothelial surface of the DEC has been addressed since the beginning of the DMEK era. Matsuzawa et al. proposed two pairs of asymmetrical semicircular marks of 1.0 and 1.5 mm in diameter placed on the edge of the 7.5 mm donor graft using dermatological biopsy punches [6]. Bachmann et al. used three marks of 1.0 mm trephined asymmetrically on the edge of the 8 mm graft [7]. Bhogal et al. introduced a single triangular mark using a 30° incision knife [8]. The area removed by trephining the marks and the loss of endothelial cells associated with these methods was theoretically assessed to be 2.5% [7] to 5.8% [6]. With our technique, the estimated endothelial cell loss is around 0.3%, given that the area of one marking dot is approximately 0.02 mm^2^, and the diameter of the graft is 8.0–8.25 mm.

As the endothelial cell loss after DMEK ranges from 33.9% to 56% in the first 6 months anyway, some surgeons neglect the ECD loss associated with the punch technique [18, 19]. However, further disadvantage of the punch technique may be the danger of the DEC being hooked against intraocular structures or creation of a graft tear. This may be the case by inconvenient recipient's properties like a shallow anterior chamber and the presence of vitreous pressure [6]. The endothelial cell loss of 35% after 6 months in our study was comparable to previous studies [16, 20, 21].

The marking techniques associated with S or F letters stamped through the stromal hole require a dermatological biopsy punch [6, 10]. In contrast to this method, the air-bubble-Braille-“R”-letter requires only a 1 ml syringe and 30 G needle in order to inject an air bubble into the interface between the endothelial surfaces of the graft. Romano et al. showed an area of surrounding damage at the sites where the mark “F” was present with either high amounts of mortality or denuded areas. Furthermore, he reported that F mark induces around 0.5%–1% cell mortality of the whole tissue [22]. The creation of the Braille-“R”-letter requires only 4 dots punctuated on the stroma and hence induces smaller endothelial cell loss in comparison to F or S marks because less marking dots are needed [23] (Figure 6).

**Figure 7:**
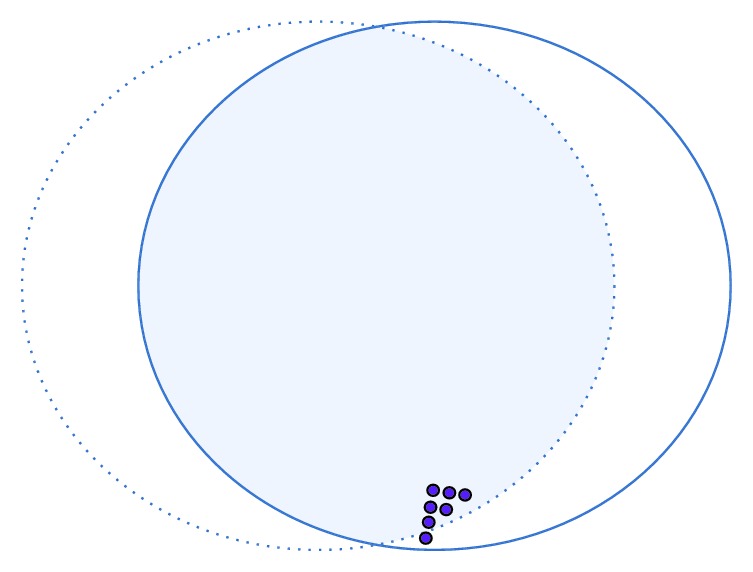
In case of an asymmetrical trephination of a graft with an “F” letter mark, the mark may become symmetrical.

In case the graft is asymmetrically trephined and the periphery cut of, the orientation of the Braille-“R”-letter can often still be identified (Figure 5(c)). We experienced that in case of an asymmetrical trephination of a graft with an “F” letter mark, the mark may become symmetrical as shown in Figure 7. Additionally, in contrast to punch techniques, the marking techniques using the stained mark or letter allow better visibility of the graft in the glass injector or in the anterior chamber.

The stripping preparation technique with a superficial trephine cut was shown to be accompanied by the lowest cell death rate in comparison to other stripping and bubble techniques. However, it is the most time-consuming and expensive preparation method [2]. The marking of the graft in our study required about 2-3 minutes. The preparation and marking of the graft did not require more time than the stripping method without marking in the study of Parekh et al. [2].

In summary, the air-bubble-Braille-“R”-letter marking technique is a simple and in some cases cost-saving supplementation of the DEC preparation to ensure correct orientation of the DEC graft intraoperatively during the DMEK procedure. The estimated loss of 0.3% of the endothelial cells is low in comparison to other marking techniques.

## Data Availability

The data used to support the findings of this study are available from the corresponding author upon request.
